# Clinical significance of pretreatment serum levels of VEGF and its receptors, IL- 8, and their prognostic value in type I and II endometrial cancer patients

**DOI:** 10.1371/journal.pone.0184576

**Published:** 2017-10-09

**Authors:** Beata Kotowicz, Malgorzata Fuksiewicz, Joanna Jonska-Gmyrek, Alicja Berezowska, Jakub Radziszewski, Mariusz Bidzinski, Maria Kowalska

**Affiliations:** 1 Laboratory of Tumor Markers, Department of Pathology and Laboratory Diagnostics, Maria Sklodowska - Curie Institute – Oncology Center, Warsaw, Poland; 2 Department of Urooncology, Maria Sklodowska - Curie Institute – Oncology Center, Warsaw, Poland; 3 Department of General, Vascular and Oncologic Surgery, Multidisciplinary Hospital, Warsaw-Miedzylesie, Poland; 4 Department of Gynecological Oncology, Maria Sklodowska - Curie Institute – Oncology Center, Warsaw, Poland; National Cancer Center, JAPAN

## Abstract

**Objectives:**

The study aimed to assess the usefulness of the determination of cytokines: IL-8, VEGF and its soluble receptors: VEGF-R1, VEGF-R2 in patients with endometrial cancer (EC).

**Material/Methods:**

The study group consisted of 118 patients with EC subjected to surgical treatment. Before the treatment we determined the serum levels of cytokines IL-8, and VEGF as well as VEGFR1 and VEGFR2 receptors. For comparison, the concentration of CA 125 was also measured. VEGFR1 and CA 125 were determined in the COBAS e601 system using Roche Diagnostics kits, while IL-8, VEGF and VEGFR2 were measured by ELISA assay using R&D Systems kits.

**Results:**

The concentrations of IL-8, VEGF, VEGFR1 and CA 125 allowed to distinguish patients for the control group. The highest diagnostic sensitivity has been shown for the concentrations of VEGF (AUC = 0.904) and IL-8 (AUC = 0.818). Among all studied parameters only CA125 concentrations increased with the clinical stage; being significantly higher in patients in FIGO III-IV, than FIGO I-IB. In patients at the FIGO stage I-IB, complementary determinations of CA 125 and VEGF resulted in the largest increase of diagnostic sensitivity. Patients with metastases to the para-aortic lymph nodes had significantly higher levels of VEGF compared to subjects without such lesions. The concentrations of IL-8 were an independent prognostic factor in the assessment of overall survival in patients with type I endometrial cancer, while the concentrations of VEGFR2 in those with type II.

**Conclusions:**

In patients with endometrial cancer, the clinical usefulness of IL-8 and VEGFR2 measurements as the potential prognostic factors has been demonstrated. In type I, the concentrations of IL-8 determined before treatment can be helpful in predicting overall survival. In patients qualified to type II EC, the concentrations of VEGFR2 have the value of an independent prognostic factor for overall survival, this requires research on larger groups of patients. The increased levels of VEGF may be useful in the preoperative assessment of the status of para-aortic lymph nodes.

## Introduction

Endometrial cancer (EC) ranks sixth regarding the incidence of malignant neoplasia among women, but it is one of the most common gynecological cancers. In most cases, it is diagnosed at an early clinical stage. It often involves patients burdened with other coexisting diseases, such as hypertension, diabetes or obesity [1]. The basic method of EC treatment is surgery—radical hysterectomy (RH) with the removal of the uterus with appendages. In some cases surgical treatment is extended by bilateral ilio-obturator lymphadenectomy. In the cases suspected of metastases to the para-aortic lymph nodes, the para-aortal lymph nodes biopsy is also performed. The final evaluation of the clinical stage is based on the examination of the postoperative material collected from the removed tissue. Due to the clinical characteristics of the patients concerned, the risk of complications resulting from the need to apply appropriate therapeutic method is a very important issue. It is relevant to distinguish the group of patients in whom lymphadenectomy is necessary. Sometimes, due to the presence of risk factors, extensive surgery is a threat, so it is reasonable to seek additional parameters that can be helpful in selecting patients for whom it is advisable to use another method of surgical treatment. Identification of factors that have an influence on the course of the disease is relevant, both for prognosis and determining the need for proper adjuvant treatment in these patients [2–5].

Based on the literature and our own studies we know that cytokines can be helpful, not only to determine the clinical stage, but primarily to predict the further course of the disease, already at early clinical stages [6–8]. Particular importance is attached to cytokines involved in the process of angiogenesis, conditioning disease progression. Among angiogenic cytokines, a key role is played by vascular endothelial growth factor (VEGF). Therefore, the use of drugs which block the activity of this factor is important in neoplastic therapy. So far, several isoforms of VEGF molecules have been identified, primarily including the angiogenic activity of VEGF-A, which is a ligand for the two receptors: VEGFR1 (sFLT1) and VEGFR2, while VEGF-C and VEGF-D are ligands for the VEGFR3 receptor. Under normal conditions, these receptors are expressed in endothelial cells; their overexpression has been observed in many types of neoplasms, which may indicate their involvement in pathological angiogenesis [9–13]. Interleukin-8 (IL-8) as the neutrophil chemoattractant stimulant also participates in tumour angiogenesis, including endometrial cancer [14–16]. Cytokines and their soluble receptors, which, due to their limited specificity of increased release in acute and chronic inflammation, in wound healing and extensive trauma, do not meet the criteria for classic tumor markers. They may be a factor characterizing the biology of tumors, useful in particular in predicting the further course of the disease.

The study aimed to determine the usefulness of the determination of cytokines: IL-8, VEGF and its soluble receptors: VEGF-R1, VEGF-R2 in patients with endometrial cancer in the preoperative prognosis of the disease. This was done by selecting the parameters of the highest diagnostic sensitivity, analyzing the relationship between the concentrations and clinicopathological features and evaluating the prognostic value of biomarkers.

## Materials and methods

The study enrolled 118 patients with histologically confirmed endometrial cancer treated at a single Center, the Maria Sklodowska-Curie Institute-Oncology Center in Warsaw, in the years 2007 to 2010. The patients' age ranged from 29 to 84 years (median age was 63 years), including 10 women before and 108 after menopause. All patients received surgical treatment—the removal of the uterus with appendages. The clinical stage of cancer according to the FIGO classification and the lymph node status were determined based on the surgical-pathological protocol of the material obtained during surgery. According to the actual recommendation for endometrial cancer treatment (ESMO, ESGO guidelines), IL8 and VEGF are not the standard indicators for an additional adjuvant treatment. Patients in an early stage of endometrial cancer were qualified to the adjuvant therapy by presence or absence of certain risk factors, as deep myometrial invasion, older age, high grade of the tumor, lymphovascular space invasion, large tumor size. Patients were qualified to low-, intermediate- and high-risk group. To the observation only, were qualified patients with no-risk or low-risk factors. Patients with risk-factors, as deep stromal infiltration, high grade, lymph vascular space invasion, older age, <50% myometrial infiltration, were qualified to vaginal brachytherapy or pelvic radiotherapy. Patients with risk-factors, as age >60 years, >50% myometrial infiltration and grade 1–2, cervical glandular involvement, grade 3 and <50% myometrial invasion (high-intermediate risk factors), were qualified to the radiotherapy on the pelvic area. Patients with metastatic lymph nodes were qualified to radiochemotherapy or chemotherapy. Patients with cancer type II, as serous, clear cell, solid, papillary, were qualified to the brachytherapy and chemotherapy. During the study, the clinical condition of patients was evaluated based on the standard gynaecological examination, ultrasound or CT of the abdomen and pelvis, chest X-ray as well as the serum CA 125 concentration. In case of suspected relapse, we performed a biopsy of lesions followed by the histopathological assessment. Written informed consent was obtained from all patients before the treatment.

The study received the approval of the Ethical Committee of the Maria Sklodowska—Curie Institute—Oncology Center in Warsaw. The approval No. 26/2005.

The clinicopathologic characteristics of the study group are shown in Table 1.

**Table 1:** Clinicopathological characteristics of ^a^EC patients.

Parameters	^b^NP	Percentage of patients
**Age**		
< 50 age /premenopausal/	10/118	8
≥ 50 age /postmenopausal/	108/118	92
^c^**FIGO stage**		
IA-IB	73/118	62
II	17/118	15
IIIA-IIIC2	19/118	16
IVA-IVB	5/118	4
^d^ND	4/118	3
**Tumor differentiation** ^e^**/G/**		
G1	34/118	29
G2	67/118	57
G3	14/118	12
Gx	3/118	2
**Pelvic lymph nodes status**		
^f^N0	101/118	86
^g^N1	16/118	13
^d^ND	1/118	1
**Tumor histopathology**		
Type I (endometrioid)	94/118	80
Type II (serous, clear cell, solid, papillary, other)	22/118	19
^d^ND	2/118	1
**Clinical status**		
^h^NED	82/118	69
^i^DR	29/118	25
Alive	83/118	70
Dead	28/118	24
^d^ND	7/118	6

Abbreviations:

^a^Endometrial cancer.

^b^Number of patients.

^c^International Federation of Gynecology and Obstetrics.

^d^No data.

^e^Grade.

^f^No metastases.

^g^Metastases.

^h^No evidence of disease.

^i^Disease recurrence.

In the study group the serum levels of cytokine IL-8 and its receptors VEGF: VEGFR1 and VEGFR2 were determined before treatment. In order to standardize clotting conditions, all sera were separated within 1 h after blood collection and stored in −70°C until assayed. The standard CA 125 marker was also measured. The concentrations of VEGFR1 (sFLT1) and CA 125 were tested by the electrochemiluminescence method in the COBAS e601 system using Roche Diagnostics kits, while the levels of IL-8, VEGF and VEGFR2 were measured by ELISA using R&D Systems kits. The control group was recruited from among the staff of the Institute. On the day of blood collection, there were no symptoms of infection in selected women. Forty nine women, aged 32 to 63 years (median 55 years) were enrolled in the control group. The sample preparation was the same as for the study group. Based on the obtained values of determined parameters concentrations, the cytokine cut-off value was determined. There was no evidence of the age effect on the level of evaluated parameters in both the patients and the control group. Standards for VEGF, VEGFR1 and VEGFR2 were developed based on the measurement of these parameters in the control group, taking 95 percentile as a cut-off. The cut-off point is the concentration value of a particular parameter that differentiates healthy and people with a disease. Standard for IL-8 was established in our previous studies [6]. Sensitivity of the method and the cut-off for CA 125 were adopted according to the kit manufacturer's recommendations. The concentration of serum levels and cut-off points of all studied parameters of healthy subject are presented in Table 2.

**Table 2:** The concentration of serum levels and cut-off points of cytokine and soluble cytokine receptor in healthy subjects.

Cytokine/Marker	Range	Median	95 percentyl cut-off
**IL-8 pg/mL**	0.5–12.7	0.5	12.5
**VEGF pg/mL**	9.0–331.0	91.0	325.0
**VEGFR1 pg/mL**	57.1–95.5	77.0	96.4
**VEGFR2 pg/mL**	6040–13864	9882	11832
**CA 125 IU/mL**	2.8–31.5	10.7	35.0*

*standard cut-off.

Statistical calculations were based on the Statistica PL 6.0 program for Windows and Excel 7.0 for Windows. The study used the Mann-Whitney test, Kruskal-Wallis test and Spearman's rank correlation coefficient. The analysis of diagnostic power for determined parameters was made using the MedCalc statistical program and included determination of the areas under the ROC curves (AUC—area under curve). The probability of disease-free survival (DFS) and overall survival (OS) was evaluated by univariate analysis using the log-rank test and Cox regression model. The borderline level of significance was adopted at P <0.05.

## Results

**Fig 1:**
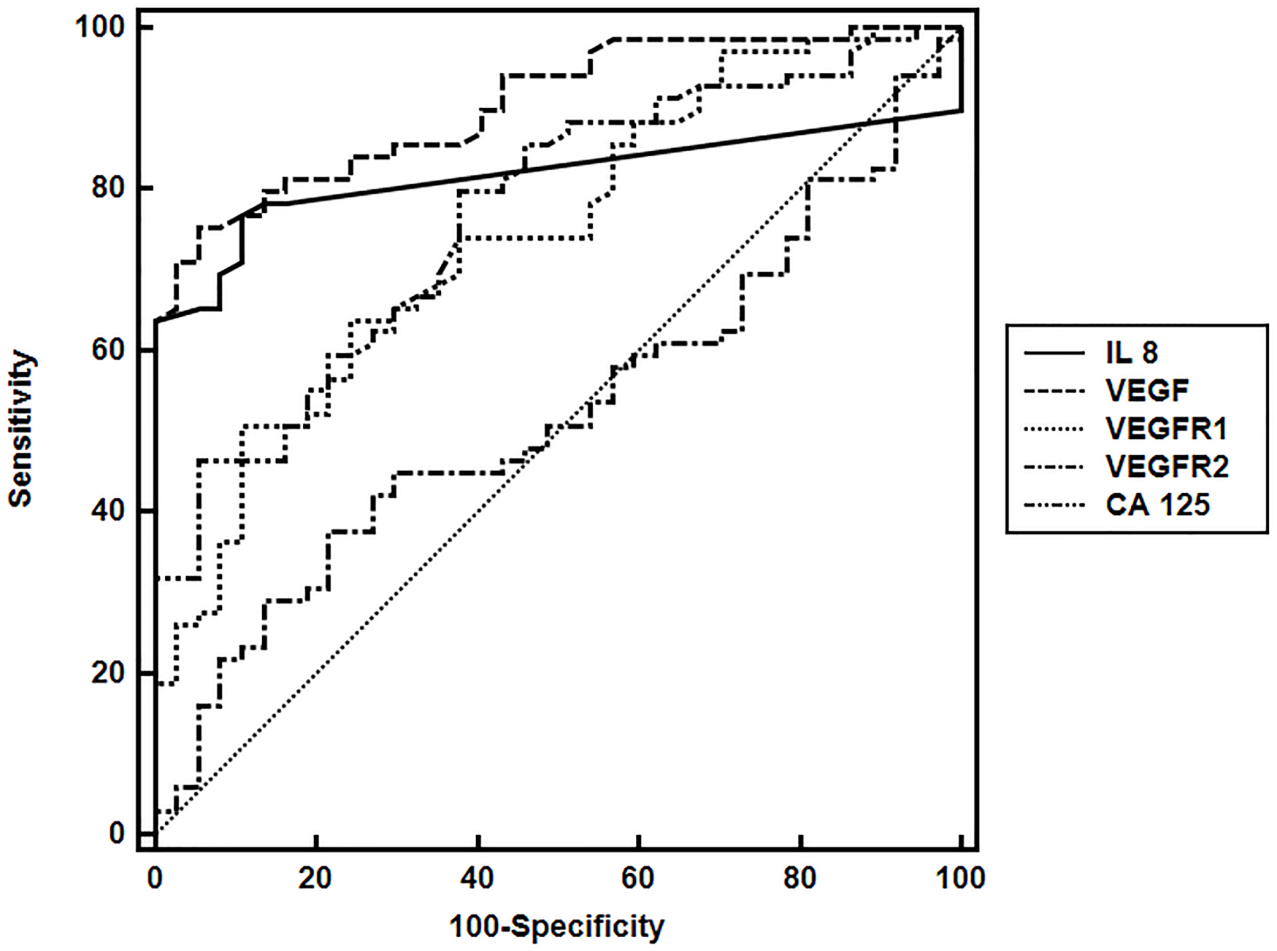
Potential of IL-8, VEGF, VEGFR1, VEGFR2, and CA 125 serum concentrations to discriminate between endometrial cancer patients and control group based on the area under the curve (AUC).

In the study group, IL-8 (65%) and VEGF (63%) concentrations were elevated most often, while the concentration of standard CA 125 marker was higher in only 29% of patients. The concentration of soluble receptors: VEGFR1 and VEGFR2 were raised in 25% and 22% of subjects, respectively. The Mann-Whitney test showed significantly higher concentrations of IL-8, VEGF, VEGFR1 and CA 125 in diseased compared to the control group, at a significance level P <0.0001. We observed elevated levels of VGFR2 in 22% of patients, but the differences in median concentrations were negligible in patients = 9897.8 pg/mL; in control group = 9882.2 pg/mL. Therefore no statistically significant differences were found between the VEGFR2 values in patients vs. control group (P = 0.558). The analysis based on ROC curves conducted in patients with endometrial cancer revealed the highest diagnostic sensitivity for the concentrations of VEGF (AUC = 0.904) and IL-8 (AUC = 0.818). The diagnostic sensitivity for VEGFR1 and VEGFR2 and comparatively for CA 125 was respectively: AUC = 0.743, 0.524 and 0.764 (Fig 1).

There were significant differences between the fields under the ROC curves for CA 125 and VEGF (P <0.007). Also, significant differences were confirmed between fields under the ROC curves; for AUC: VEGF and VEGF R1 (P<0.003); VEGF and VEGF R2 (P<0.001) and between VEGFR1 and VEGFR2 (P<0.005). Similar results were obtained in the ROC analysis performed in patients at the early stages of the disease, i.e. FIGO I-IB; the highest diagnostic sensitivity has been shown for VEGF (AUC = 0.917) and IL-8 (AUC = 0.841).

The assessment of the relationship between the concentrations of determined parameters and the clinical stage: FIGO I-IB vs. II vs. III + IVB showed that with the progression of the disease the proportion of patients with the elevated levels of standard marker CA125 increased. However, the relationship was observed only for this marker. The concentrations of cytokines: IL-8 and VEGF were elevated in a high proportion of patients, both at the early (49% and 64%) and advanced stages of the disease (58% and 57%). The Kruskal-Wallis test and Spearman rank correlation coefficient demonstrated that only the concentrations of CA 125 (P <0.0001, R = 0.39) grew with the clinical FIGO stage (Table 3).

**Table 3:** The value of medians, statistical differences and the percentage of patients with elevated levels of CA 125 and cytokines, in dependence on the clinical stage.

Marker/Cytokine	I-IB (n = 73)		II (n = 17)		IIIA-IVB (n = 24)		p
	Median/range	%	Median/range	%	Median/range	%	
**CA 125 IU/mL**	14.9 5.6–147	12	24.8 9.6–215	35	38.5 4.0–523	58	0.0001
**IL-8 pg/mL**	12.5 4.1–32.6	49	11.0 5.0–28.0	35	13.2 7.7–289	58	NS*
**VEGF pg/mL**	510 78–884	64	429.7 74–864	56	526.5 78–1069	57	NS*
**VEGF R1 pg/mL**	84.2 60,6–106	19	88.5 59.1–114	29	87.9 70.0–124	29	NS*
**VEGF R2 pg/mL**	9778 6813–12103	20	11057 6006–11892	10	10948 7536–16878	33	NS*

*NS—not statistically significant.

**Fig 2:**
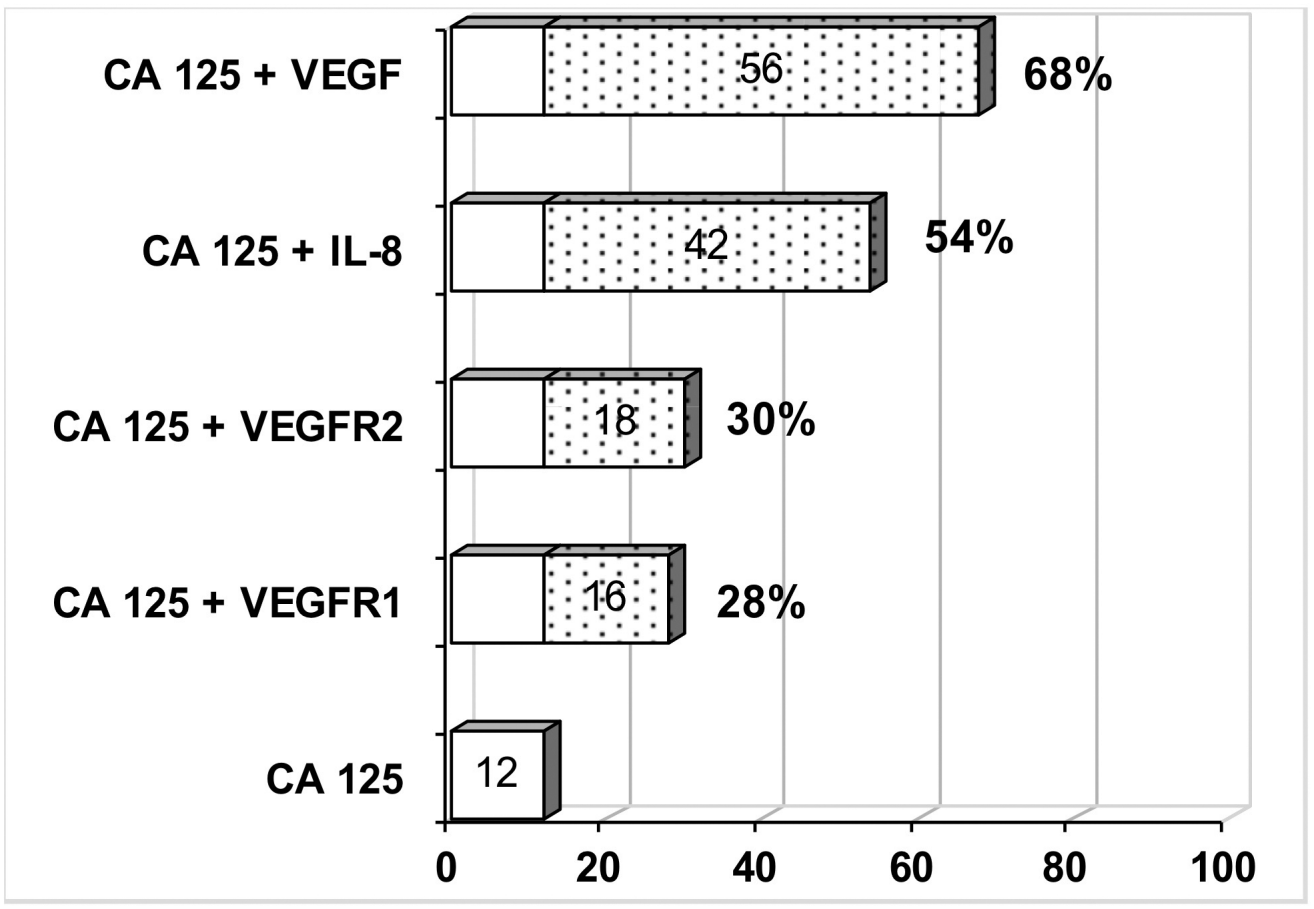
The diagnostic sensitivity of the complementary evaluation of CA 125 with: VEGF, IL-8, VEGF R2 and VEGF R1 in endometrial cancer patients in FIGO stage I-IB.

The next stage evaluated which of the determined parameters can increase the low diagnostic sensitivity of the standard CA 125 marker, particularly in patients at the early stages of clinical progression. In 73 patients with early FIGO stage (I—IB), the diagnostic sensitivity of CA 125 was only 12%. In the remaining patients in the analyzed group, where CA125 concentrations were below the cut-off point, further tests of marker and cytokines together with CA 125 were investigated to increase diagnostic sensitivity. As a result of this analysis, we found that by identifying CA125 with IL8 the sensitivity increased by 54%, the sensitivity of complementary determination of CA125 and VEGF has grown up to 68%. Fig 2 presents the results of the complementary determination of CA 125 with the remaining parameters tested.

**Fig 3:**
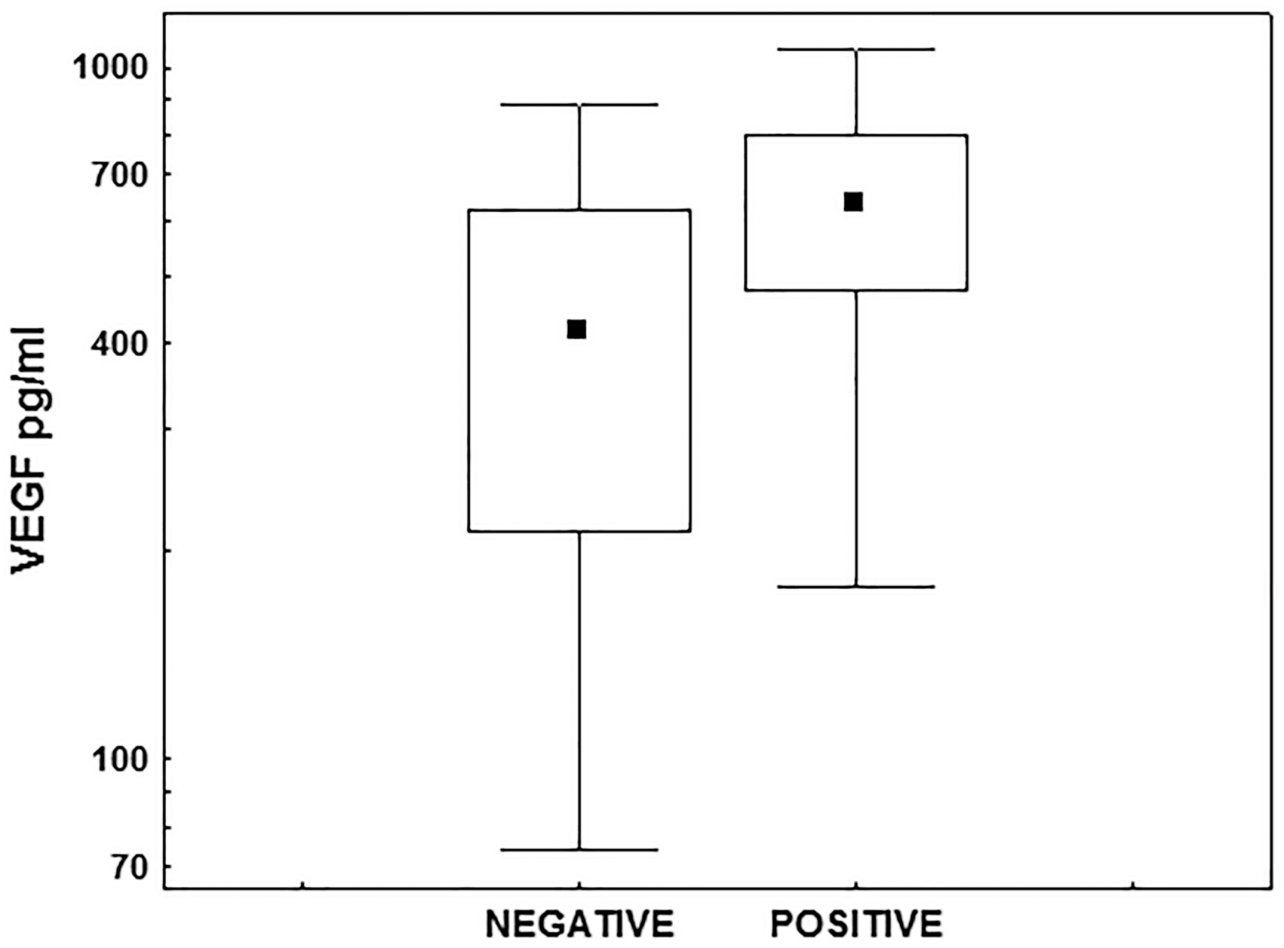
Medians and VEGF concentrations in dependence on regional lymph nodes status.

Seeking the relationship between standard CA 125 marker and determined cytokines, using the Spearman rank test, we found the correlation between the concentrations of CA125 and IL 8 (P <0.043; R = 0.19). We did not demonstrate such correlation between serum levels of VEGF and its receptors. Then, the relationship was analyzed between the concentrations of biomarkers determined before treatment and clinicopathological features, such as tumour histology grade (G), the state of the regional lymph nodes and tumour type (type I/II). Ilio-obturator lymphadenectomy was performed in 114 patients, metastases were found in 13%. A particular type of cancer was identified based on the histopathological examination of the postoperative material; type I—in 94 patients and type II—in 22 patients. Among studied cytokines, the significant relationships were found only between the concentrations of VEGF and the presence of metastases in the para-aortic lymph nodes. In patients with metastases (N1), the concentrations of VEGF were significantly higher (P <0.019) than in subjects without theses lesions (N0) (Fig 3). The medians and the ranges of VEGF levels in both patient groups were as follow: N0 group; median 418.5 (74–884) pg/ml, N1 group; median 641.0 (191–1069) pg/mL.

There was no statistical relationship between the concentrations of the tested parameters, the type of neoplasm and a degree of malignancy (G).

The most important stage of the study evaluated the relationship between the concentrations of biomarkers, clinico-pathological parameters and disease-free survival (DFS) and overall survival (OS). During the 5-year follow-up (median follow-up was 1935 days), among 118 subjects, 82 remained in remission, in 29 (25%) the disease progressed, in 28 (24%) observation gave fatal outcomes, the clinical status could not be determined in 7 patients. The Mann-Whitney test demonstrated that in patients who relapsed, the concentrations of IL-8 (P <0.011), VEGF (p <0.044) and CA 125 (P <0.015) were significantly higher compared to those without recurrence. Of all studied biomarkers, only the levels of IL-8 (P <0.002) were significantly higher in patients who died during follow-up compared to the group of alive women. The relationship was assessed between DFS and the concentrations of parameters determined before treatment in the entire group of patients. Clinico-pathological parameters, as FIGO stage (P <0.003), tumor type (type/II) (P <0.0001) and the status of regional lymph nodes (P <0.002) were confirmed as prognostic factors for DFS.

**Fig 4:**
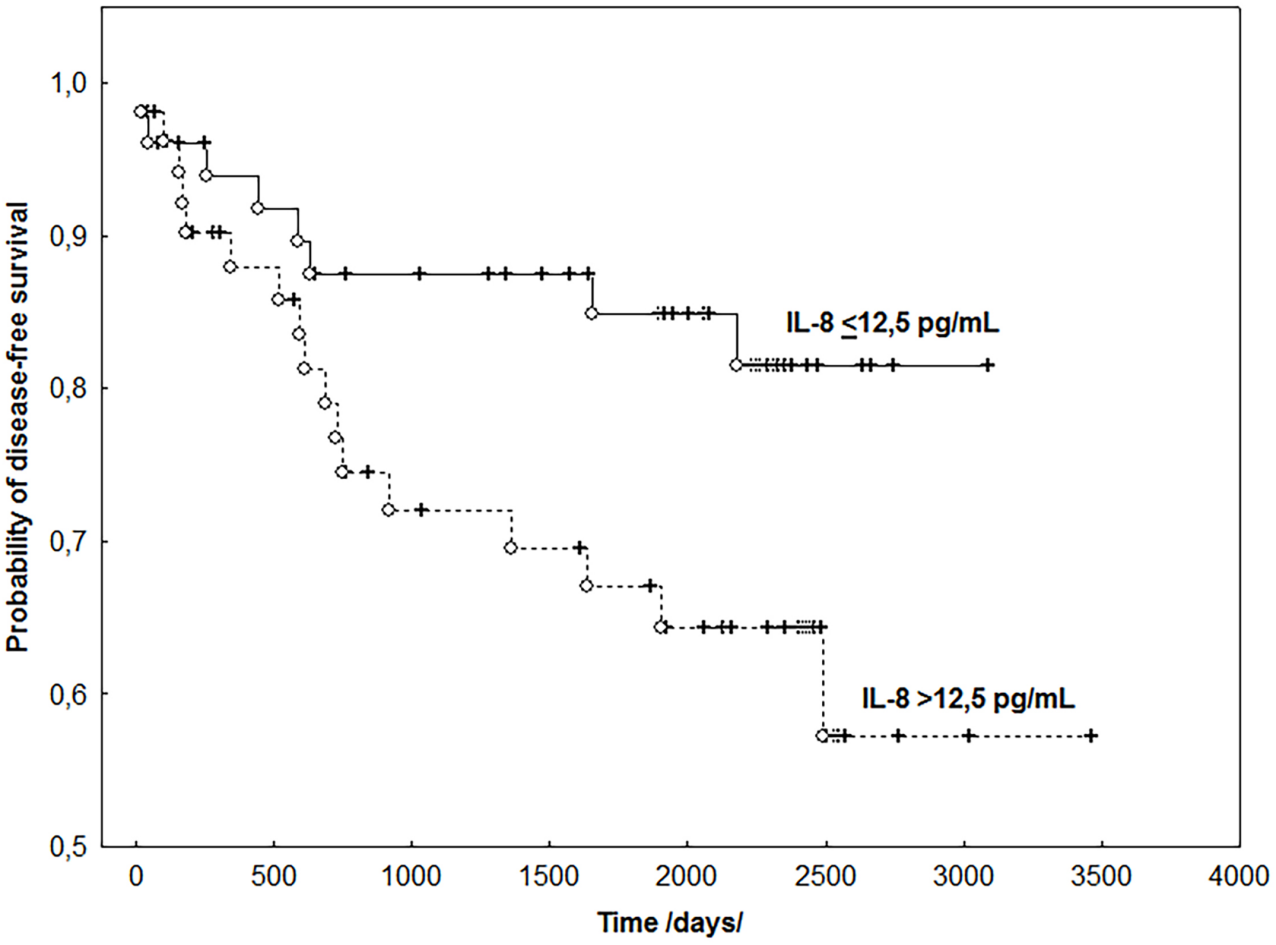
The probability of disease free survival according to the IL-8 levels in endometrial cancer patients.

Among biochemical parameters, the log-rank univariate analysis confirmed the correlation between CA125 and DFS (P <0.001). However, among the tested cytokines, the relationship was demonstrated between the elevated levels of IL-8 (P <0.048) and shorter disease-free survival (Fig 4).

**Fig 5:**
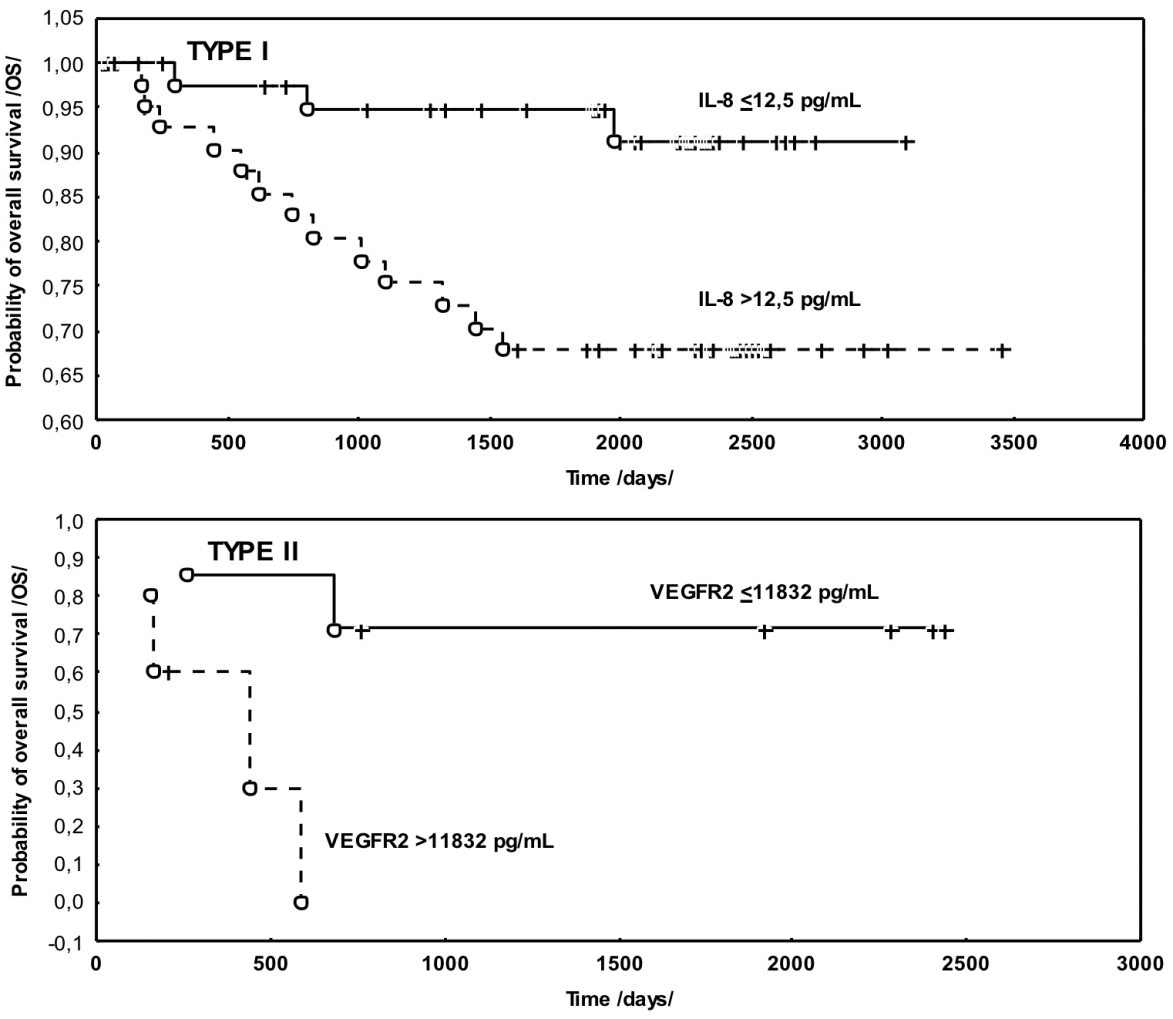
The probability of overall survival according to the IL-8 and VEGFR2 levels in endometrial cancer patients, type I/II.

The analysis of the predictive value of the examined parameters for OS conducted using the log-rank test demonstrated the prognostic value for the same clinical parameters and biomarkers: IL-8 (P <0.04) and CA 125 (P <0.004). Multivariate Cox analysis, however, confirmed the predictive value for DFS (P <0.0001) and OS (P <0.041) of only the type of endometrial cancer (I/II). Survival analysis revealed that the type of neoplasm was an independent prognostic factor both for DFS and OS, therefore, the prognostic evaluation of the tested parameters was carried out in groups of patients homogeneous in terms of this feature. Although univariate analysis revealed that in patients with type I endometrial cancer, the lymph node status (P<0.016), FIGO stage (P <0.025), the concentrations of IL-8 (P <0.009) and CA125 (P <0.030) were associated with shorter overall survival, multivariate analysis showed that only the concentration of IL-8 (P <0.026; HR: 4.253; 95% CI: 2.975–5.531) had an independent prognostic value for OS. The elevated IL-8 levels were connected with shorter OS in those patients (Fig 5).

However, in this group, no relationship was found between the assessed clinical and biochemical parameters and DFS. In patients with type II cancer, the log-rank test demonstrated the relationship only between CA 125 (P <0.013), histological grade G (P < 0.017) and DFS. This was not confirmed in the Cox analysis. Overall survival of these patients, however, depended on the state of the lymph nodes (P <0.025), the concentration of VEGFR2 (P <0.021) and CA 125 (P <0.030). Nevertheless, only the concentration of VEGFR2 receptor (P <0.035; HR: 5.137; 95% CI: 3.614–6.660) proved to be an independent prognostic factor for OS in patients with type II endometrial cancer.

## Discussion

The process of angiogenesis plays a key role in the development of neoplasms. Under physiological conditions this phenomenon is adjustable, whereas in patients with cancer it is not subject to the control mechanisms. It leads to the increased synthesis of pro-angiogenic factors and decreased expression of its inhibitors. Vascular endothelial growth factor (VEGF) is an important pro-angiogenic element. VEGF is a glycoprotein produced by a plurality of cells, but it primarily affects endothelial cells. It plays an important role in normal and pathological angiogenesis [11, 17]. The activity of cytokines depends on the intensity of the expression of specific receptors present on target cells. It is only upon binding to a cognate receptor, whose extramembranous portion is located at the surface of cells, cytokines can have an biological effect. The presence of VEGF receptors, referred to as VEGFR-1 and VEGFR-2, has been demonstrated in endothelial cells; their overexpression has been observed in several types of neoplasms. This may also indicate their participation in pathological angiogenesis [9, 12, 18]. Interleukin-8 affects tumour angiogenesis by stimulation of chemotaxis, proliferation and migration of endothelial cells [15, 16, 19]. Pro-angiogenic cytokines are the subject of intensive research, their role as potential biomarkers is evaluated, especially in the context of their prognostic significance. Neoplasms of reproductive organs, including EC are accompanied by the elevated levels of pro-angiogenic factors, which may be associated with poor prognosis [18–21].

Like other studies, the first stage of our investigation demonstrated significantly higher levels of VEGF, IL8 and VEGFR1 in the group of patients compared with healthy women [20, 22]. We did not show such a relationship for VEGFR2 concentrations. Koukourakis et al. noted the increased expression of the VEGFR2 receptor in patients with endometrial cancer, breast cancer and ovarian cancer, accompanied by the elevated serum levels of VEGF [20]. Further, our study conducted in patients with low levels of clinical staging (FIGO IB-I) reported a higher diagnostic sensitivity of determined cytokines compared to standard CA 125 marker. An interesting stage of our work was to demonstrate that the complementary determination of CA 125 marker, VEGF and IL-8 in patients with the FIGO stages I-IB can produce a significant increase in its diagnostic sensitivity. There were no similar works found in the available literature. ROC analysis used in our study also confirmed the highest diagnostic sensitivity for the concentrations of VEGF and IL-8. The concentrations of these cytokines were elevated in a high percentage of patients, even at low degrees of staging (FIGO IB-I). However, just like Powell JL et al., we have shown a significant correlation only between CA125 marker and the FIGO stage [23]. We have also found a statistical correlation between the concentrations of cytokine IL-8 and marker CA 125.

Most importantly, our studies examining the relationship between the concentrations of biomarkers and other clinicopathological features, demonstrated the relationship between the concentrations of VEGF cytokine and the presence of metastases to the pelvic minor lymph nodes. There was no such a relationship observed for the concentrations of VEGFR1 and VEGFR2. Sami K. et al. obtained similar results, indicating significantly higher levels of VEGF in patients with metastases, and no statistical relationships for the VEGFR1 concentrations [18]. Like other authors, we did not demonstrate the relationship between the concentrations of cytokines, their receptors and histological grade (G).

The most important stage of our work was to evaluate the prognostic value of the examined parameters, which was made possible by about 5 years of follow-up conducted in 118 patients with EC. Even though, in addition to clinicopathological features, the log-rank univariate analysis has shown the correlation between the concentrations of cytokine IL-8 and both DFS and OS, Cox multivariate analysis indicated a type of neoplasm to be an independent prognostic factor in our group of patients. Therefore, it was important to examine whether in a group homogeneous in terms of the type of neoplasm determined biomarkers could have a prognostic value. Our studies have shown that in patients with type I tumour, out of all clinical and biochemical parameters, interleukin-8 was a poor independent prognostic factor for OS whose elevated levels were associated with shorter OS. We have not found in the available literature works concerning determination of IL-8 in patients with EC. Examining the relationship between the concentrations of IL-8 and abdominal obesity, only Ciortea R, et al. demonstrated the significantly higher levels of this cytokine in patients with EC compared with the control group [21]. Although the key pro-angiogenic cytokines, especially VEGF, for years have been the subject of many studies, IL-8 as an angiogenesis promoting agent is very interesting and requires further investigation. Understanding pro-angiogenic factors, exponents of increased neovascularisation, is the basis for research focused on the new ways of treating neoplasms—anti-angiogenic therapy.

Survival analysis gave interesting results in patients who have had far less common, but more aggressive, type II cancer. Our studies showed that in this group of patients the concentrations of VEGF receptor, VEGFR2 proved to be an independent prognostic factor for OS. Most recent literature data presents results of studies on the relationship between the expression of VEGF, its receptors and recognized clinicopathological factors, and their role in the prognosis of the disease [24, 25]. Among numerous analyzed clinicopathological features, the authors have observed only a trend of relationship between the expression of VEGFR2 and the degree of cell differentiation (G), but no association was shown between the expression of VEGF-R2 and survival of patients with EC, however, not taking into account the type of neoplasm [26]. It should be stressed that our results relate to determinations of serum VEGFR2 in the selected group of patients with diagnosed type II EC. From the clinical point of view, it was particularly interesting to prove in our study the relationship between the concentrations of VEGF receptor 2 and survival of patients. The second soluble receptor, which by binding with VEGF may modify angiogenic activity, proved to have the prognostic value. However, a small number of patients with type II, less common, but associated with poor prognosis, poses a difficulty in the interpretation of the results. It should be emphasized that VEGFR2 is responsible for starting the formation of new blood vessels, their permeability, division and promoting cell survival. This receptor is one of the points of the targeted therapy holders responsible for catching antibodies approved by the FDA for the treatment of neoplastic patients (e.g. Ramucirumab) [27].

## Conclusion

Among the studied parameters, the concentrations of VEGF and IL-8 have been shown to have the highest diagnostic sensitivity in patients with EC. In subjects at the early stages of the disease, the complementary determination of standard CA 125 marker, VEGF and IL-8 significantly increased the sensitivity of diagnostic tests. Among the analyzed clinicopathological parameters, tumour type (type I/II) is an independent prognostic factor for DFS and OS. However, the elevated levels of IL-8 are associated with shorter DFS and OS. In a homogeneous group of patients (type I EC), the elevated levels of IL-8 before treatment are a poor independent prognostic factor for OS. VEGFR2 receptor concentrations in patients with type II tumour turned out to be an independent prognostic factor for OS, although this requires confirmation in a larger group of patients.

## Supporting information

S1 File 1-endometrioid; 2-serous; 3-clear cell; 4-solid, non-differentiated; 5 papillary; 6- other, Reccurence 1-yes/2-no, Death 1-yes 2-alive. (XLS)
